# 5,6-Dichloro-2-(2-hydroxy­phen­yl)­iso­indoline-1,3-dione

**DOI:** 10.1107/S1600536808008180

**Published:** 2008-04-02

**Authors:** Orhan Büyükgüngör, Mustafa Odabaşoğlu

**Affiliations:** a Department of Physics, Faculty of Arts and Sciences, Ondokuz Mayıs University, TR-55139 Kurupelit Samsun, Turkey; b Department of Chemistry, Faculty of Arts and Sciences, Ondokuz Mayıs University, TR-55139 Kurupelit Samsun, Turkey

## Abstract

In the mol­ecule of the title compound, C_14_H_7_Cl_2_NO_3_, the phthalimide ring system is virtually planar, with a dihedral angle between the fused five- and six-membered rings of 4.02 (3)°. In the crystal structure, inter­molecular C—H⋯O and O—H⋯O hydrogen bonds and C—Cl⋯O close contacts [Cl⋯O = 3.0123 (13) Å and C—Cl⋯O = 171.14 (7)°] link the mol­ecules, generating *R*_2_^2^(16), *R*_4_^2^(19) and *R*_4_^4^(22) ring motifs by *C*(6) chains to form a three-dimensional network. A weak π–π inter­action between the six-membered rings of the phthalimide ring systems further stabilizes the structure, with a centroid–centroid distance of 3.666 (3) Å and an interplanar separation of 3.568 Å.

## Related literature

For general background, see: Chapman *et al.* (1979 ▶); Hall *et al.* (1983 ▶, 1987 ▶); Srivastava *et al.* (2001 ▶); Cechinel *et al.* (2003 ▶); Abdel-Hafez (2004 ▶); Antunes *et al.* (2003 ▶); Sena *et al.* (2007 ▶). For ring motif details, see: Bernstein *et al.* (1995 ▶); Etter (1990 ▶).
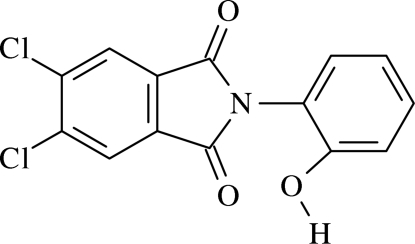


## Experimental

### 

#### Crystal data

C_14_H_7_Cl_2_NO_3_
*M* *_r_* = 308.11
Monoclinic, 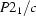

*a* = 7.5993 (2) Å
*b* = 19.4088 (5) Å
*c* = 9.5086 (3) Å
β = 110.842 (2)°
*V* = 1310.68 (7) Å^3^
*Z* = 4
Mo *K*α radiation
μ = 0.50 mm^−1^
*T* = 296 K
0.63 × 0.43 × 0.24 mm

#### Data collection

Stoe IPDSII diffractometer
Absorption correction: integration (*X-RED32*; Stoe & Cie, 2002 ▶) *T*_min_ = 0.759, *T*_max_ = 0.881
20050 measured reflections
2783 independent reflections
2341 reflections with *I* > 2σ(*I*)
*R*_int_ = 0.057

#### Refinement

*R*[*F* ^2^ > 2σ(*F* ^2^)] = 0.035
*wR*(*F* ^2^) = 0.096
*S* = 1.04
2783 reflections
182 parameters
H-atom parameters constrained
Δρ_max_ = 0.21 e Å^−3^
Δρ_min_ = −0.28 e Å^−3^

### 

Data collection: *X-AREA* (Stoe & Cie, 2002 ▶); cell refinement: *X-AREA*; data reduction: *X-RED32* (Stoe & Cie, 2002 ▶); program(s) used to solve structure: *SHELXS97* (Sheldrick, 2008 ▶); program(s) used to refine structure: *SHELXL97* (Sheldrick, 2008 ▶); molecular graphics: *ORTEP-3 for Windows* (Farrugia, 1997 ▶); software used to prepare material for publication: *WinGX* (Farrugia, 1999 ▶).

## Supplementary Material

Crystal structure: contains datablocks I. DOI: 10.1107/S1600536808008180/hk2440sup1.cif

Structure factors: contains datablocks I. DOI: 10.1107/S1600536808008180/hk2440Isup2.hkl

Additional supplementary materials:  crystallographic information; 3D view; checkCIF report

## Figures and Tables

**Table 1:** Hydrogen-bond geometry (Å, °)

*D*—H⋯*A*	*D*—H	H⋯*A*	*D*⋯*A*	*D*—H⋯*A*
O3—H3*A*⋯O2^i^	0.82	1.90	2.7235 (18)	177
C3—H3⋯O3^ii^	0.93	2.55	3.397 (2)	152
C13—H13⋯O3^iii^	0.93	2.59	3.505 (2)	168

Symmetry codes: (i) 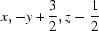; (ii) 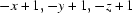; (iii) 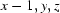.

**Fig. 1.**
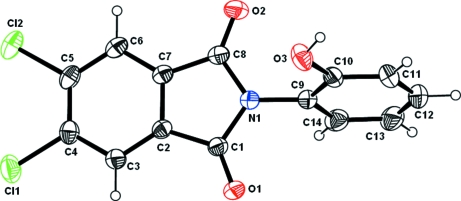
The molecular structure of the title molecule, with the atom-numbering scheme. Displacement ellipsoids are drawn at the 30% probability level.

**Fig. 2.**
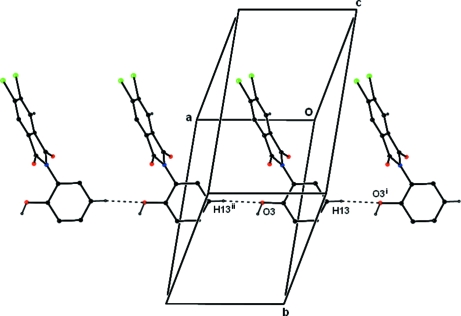
A partial packing diagram of (I), showing the formation of C(6) chain [symmetry codes: (i) x - 1, y, z; (ii) x + 1, y, z]. H atoms not involved in hydrogen bondings have been omitted for clarity.

**Fig. 3.**
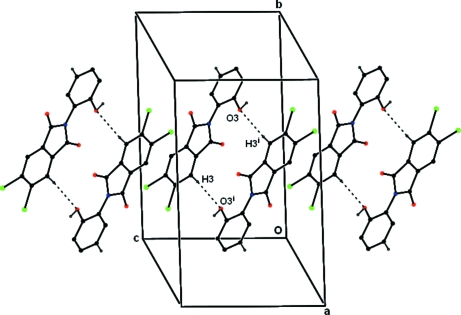
A partial packing diagram of (I), showing the formation of centro- symmetric R22(16) ring motifs. Hydrogen bonds are shown as dashed lines [symmetry code: (i) x + 1/2, 1 - y, 1 - z]. H atoms not involved in hydrogen bondings have been omitted for clarity.

**Fig. 4.**
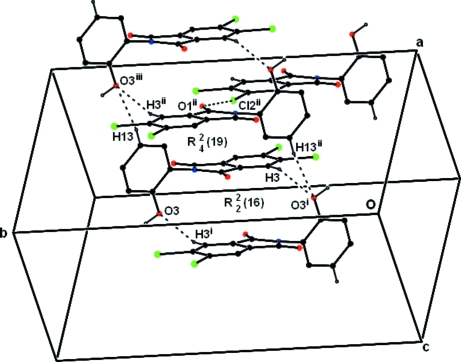
A partial packing diagram of (I), showing the formation of R22(16) and R42(19) ring motifs. Hydrogen bonds are shown as dashed lines [symmetry codes: (i) x + 1/2, 1 - y, 1 - z; (ii) -x, 1 - y, 1 - z; (iii) x - 1, y, z]. H atoms not involved in hydrogen bondings have been omitted for clarity.

**Fig. 5.**
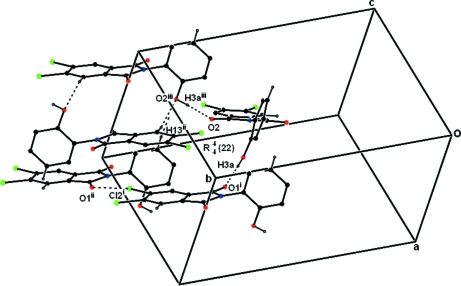
A partial packing diagram of (I), showing the formation of R44(22) ring motifs. Hydrogen bonds are shown as dashed lines [symmetry codes: (i) x, 3/2 - y, z - 1/2; (ii) x + 1, 3/2 - y, z + 1/2; (iii) x, 3/2 - y, z + 1/2].

## supplementary crystallographic information

### Comment

Phthalimide derivatives have been gaining considerable interest since 1979, when Chapman *et al.* tested the hypolipidemic activity of 23 N-substituted phthalimide derivatives (Chapman *et al.*,  1979). Later on, Hall and co-workers reported the antihyperlipidemic activity of phthalimide analogs in rodents and also the same activity was found by the administration of *ortho*-(N-phthalimido) acetophenone in sprague dawley rats (Hall *et al.*,  1983; 1987). In 2001, Srivastava *et al.* reported hypolipidemic activity in α-*D*-mannopyranosides containing phthalimidomethyl function as aglycone (Srivastava *et al.*,  2001). There are other interesting biological aspects of these compounds which have been reviewed in 2003 (Cechinel *et al.*, 2003). A recent paper cites the synthesis and anticonvulsant behavior of N-substituted phthalimides (Abdel-Hafez, 2004). Besides, certain phthalimide derivatives are synthetically important, and can be transformed to other useful products (Antunes *et al.*,  2003). In 2007, Sena *et al.* prepared ten *N*-arylaminomethyl-arylaminomethyl- and two [1,2,4-triazol-3- and 4-yl]phthalimides that these imides are potential candidates for biological evaluations (Sena *et al.*,  2007). In view of the importance of the *N*-arylphthalimides, we herein report the crystal structure of the title compound, (I).

The molecule of (I), (Fig. 1), is built up from a phthalimide unit connected to a *o*-hydroxyphenyl group through a nitrogen atom. Rings A (C2-C7), B (C1/C2/C7/C8/N1) and C (C9-C14) are, of course, planar. The dihedral angles between them are A/B = 4.02 (3)°, A/C = 75.55 (3)° and B/C = 75.13 (3)°. So, rings A and B are also nearly coplanar. Ring C is oriented with respect to the coplanar ring system at a dihedral angle of 75.37 (3)°.

In the crystal structure, intermolecular C-H···O and O-H···O hydrogen bonds (Table 1) and C-Cl···O close contacts [Cl2^i^···O1^ii^ = 3.0123 (13) Å and C5-Cl2^i^···O1^ii^ = 171.14 (7)°; symmetry codes: (i) x, 3/2 - y, z - 1/2 and (ii) x + 1, 3/2 - y, z + 1/2] link the molecules, generating R_2_^2^(16) (Fig. 3), R_4_^2^(19) (Fig. 4) and R_4_^4^(22) (Fig. 5) ring motifs by C(6) chains (Fig. 2) (Bernstein *et al.*,  1995; Etter, 1990), to form a three-dimensional network, in which they may be effective in the stabilization of the structure. A weak π···π interaction between the A rings, at x, y, z and 1 - x, 1 - y, 2 - z, further stabilizes the structure, with a centroid-centroid distance of 3.666 (3) Å and plane-plane separation of 3.568 Å.

### Experimental

A mixture of 4,5-dichlorophthalic acid (1.175 g, 5 mmol) and 2-aminophenol (0.545 g, 20 mmol) in DMF (1.5 ml) was heated at boiling temperature for 15 min, and then ethanol (50 ml, 95%) was added. Crystals of (I) suitable for X-ray analysis were obtained by slow evaporation of the mixture at room temperature (yield; 80%, m.p. 546-548 K).

### Refinement

H atoms were positioned geometrically, with O-H = 0.82 Å (for OH) and C-H = 0.93 Å for aromatic H, and constrained to ride on their parent atoms with U_iso_(H) = xU_eq_(C,O), where x = 1.5 for OH H and x = 1.2 for aromatic H atoms.

### Crystal data

**Table:**

C_14_H_7_Cl_2_NO_3_	*F*_000_ = 624
*M**_r_* = 308.11	*D*_x_ = 1.561 Mg m^−^^3^
Monoclinic, *P*2_1_/*c*	Mo *K*α radiation λ = 0.71073 Å
Hall symbol: -P 2ybc	Cell parameters from 20050 reflections
*a* = 7.5993 (2) Å	θ = 2.1–27.2º
*b* = 19.4088 (5) Å	µ = 0.50 mm^−^^1^
*c* = 9.5086 (3) Å	*T* = 296 K
β = 110.842 (2)º	Prism, light yellow
*V* = 1310.68 (7) Å^3^	0.63 × 0.43 × 0.24 mm
*Z* = 4	

### Data collection

**Table:**

Stoe IPDSII diffractometer	2783 independent reflections
Monochromator: plane graphite	2341 reflections with *I* > 2σ(*I*)
Detector resolution: 6.67 pixels mm^-1^	*R*_int_ = 0.057
*T* = 296 K	θ_max_ = 26.7º
w–scan rotation method	θ_min_ = 2.1º
Absorption correction: integration(X-RED32; Stoe & Cie, 2002)	*h* = −9→9
*T*_min_ = 0.759, *T*_max_ = 0.881	*k* = −24→24
20050 measured reflections	*l* = −12→12

### Refinement

**Table:**

Refinement on *F*^2^	Secondary atom site location: difference Fourier map
Least-squares matrix: full	Hydrogen site location: inferred from neighbouring sites
*R*[*F*^2^ > 2σ(*F*^2^)] = 0.035	H-atom parameters constrained
*wR*(*F*^2^) = 0.096	*w* = 1/[σ^2^(*F*_o_^2^) + (0.0491*P*)^2^ + 0.2555*P*] where *P* = (*F*_o_^2^ + 2*F*_c_^2^)/3
*S* = 1.04	(Δ/σ)_max_ = 0.001
2783 reflections	Δρ_max_ = 0.21 e Å^−^^3^
182 parameters	Δρ_min_ = −0.28 e Å^−^^3^
Primary atom site location: structure-invariant direct methods	Extinction correction: none

### Special details

**Table:**

Geometry. All e.s.d.'s (except the e.s.d. in the dihedral angle between two l.s. planes) are estimated using the full covariance matrix. The cell e.s.d.'s are taken into account individually in the estimation of e.s.d.'s in distances, angles and torsion angles; correlations between e.s.d.'s in cell parameters are only used when they are defined by crystal symmetry. An approximate (isotropic) treatment of cell e.s.d.'s is used for estimating e.s.d.'s involving l.s. planes.
Refinement. Refinement of F^2^ against ALL reflections. The weighted R-factor wR and goodness of fit S are based on F^2^, conventional R-factors R are based on F, with F set to zero for negative F^2^. The threshold expression of F^2^ > 2sigma(F^2^) is used only for calculating R-factors(gt) etc. and is not relevant to the choice of reflections for refinement. R-factors based on F^2^ are statistically about twice as large as those based on F, and R- factors based on ALL data will be even larger.

### Fractional atomic coordinates and isotropic or equivalent isotropic displacement parameters (Å^2^)

**Table:**

	*x*	*y*	*z*	*U*_iso_*/*U*_eq_	
Cl1	0.80274 (8)	0.37446 (3)	1.02115 (6)	0.07009 (18)	
Cl2	0.94040 (7)	0.51106 (3)	1.20351 (5)	0.06418 (17)	
O1	0.26937 (18)	0.49684 (6)	0.49490 (13)	0.0517 (3)	
O2	0.47116 (19)	0.69592 (7)	0.75853 (16)	0.0587 (3)	
O3	0.40599 (18)	0.69007 (7)	0.39975 (18)	0.0624 (4)	
H3A	0.4209	0.7246	0.3554	0.094*	
N1	0.33154 (18)	0.60578 (7)	0.60028 (15)	0.0420 (3)	
C1	0.3563 (2)	0.53405 (8)	0.59612 (17)	0.0398 (3)	
C2	0.5079 (2)	0.51681 (8)	0.74085 (17)	0.0392 (3)	
C3	0.5789 (2)	0.45386 (9)	0.79893 (18)	0.0458 (4)	
H3	0.5389	0.4135	0.7440	0.055*	
C4	0.7133 (2)	0.45279 (10)	0.94342 (19)	0.0471 (4)	
C5	0.7747 (2)	0.51343 (10)	1.02414 (18)	0.0480 (4)	
C6	0.7042 (2)	0.57666 (10)	0.96318 (18)	0.0482 (4)	
H6	0.7463	0.6173	1.0162	0.058*	
C7	0.5688 (2)	0.57720 (9)	0.82043 (17)	0.0411 (3)	
C8	0.4593 (2)	0.63491 (9)	0.73015 (19)	0.0433 (4)	
C9	0.1835 (2)	0.64372 (8)	0.49155 (18)	0.0424 (4)	
C10	0.2248 (2)	0.68665 (9)	0.3911 (2)	0.0467 (4)	
C11	0.0805 (3)	0.72369 (10)	0.2877 (2)	0.0589 (5)	
H11	0.1058	0.7522	0.2185	0.071*	
C12	−0.1002 (3)	0.71844 (11)	0.2873 (3)	0.0682 (6)	
H12	−0.1962	0.7440	0.2187	0.082*	
C13	−0.1400 (3)	0.67573 (12)	0.3875 (3)	0.0683 (6)	
H13	−0.2625	0.6724	0.3864	0.082*	
C14	0.0020 (3)	0.63797 (11)	0.4894 (2)	0.0562 (5)	
H14	−0.0247	0.6087	0.5565	0.067*	

### Atomic displacement parameters (Å^2^)

**Table:**

	*U*^11^	*U*^22^	*U*^33^	*U*^12^	*U*^13^	*U*^23^
Cl1	0.0715 (3)	0.0663 (3)	0.0640 (3)	0.0148 (2)	0.0136 (2)	0.0208 (2)
Cl2	0.0489 (2)	0.0998 (4)	0.0362 (2)	0.0005 (2)	0.00566 (17)	0.0018 (2)
O1	0.0567 (7)	0.0453 (7)	0.0417 (6)	−0.0052 (5)	0.0037 (5)	−0.0066 (5)
O2	0.0618 (8)	0.0423 (7)	0.0678 (8)	−0.0033 (6)	0.0181 (6)	−0.0142 (6)
O3	0.0524 (7)	0.0550 (8)	0.0860 (10)	0.0091 (6)	0.0323 (7)	0.0242 (7)
N1	0.0434 (7)	0.0367 (7)	0.0412 (7)	−0.0020 (5)	0.0095 (6)	−0.0002 (5)
C1	0.0419 (8)	0.0395 (8)	0.0372 (8)	−0.0037 (6)	0.0131 (6)	−0.0015 (6)
C2	0.0397 (7)	0.0433 (8)	0.0339 (7)	−0.0042 (6)	0.0122 (6)	−0.0022 (6)
C3	0.0488 (9)	0.0427 (9)	0.0434 (8)	−0.0011 (7)	0.0134 (7)	−0.0002 (7)
C4	0.0448 (8)	0.0557 (10)	0.0418 (8)	0.0044 (7)	0.0165 (7)	0.0089 (7)
C5	0.0398 (8)	0.0694 (12)	0.0341 (7)	−0.0010 (8)	0.0122 (6)	−0.0010 (8)
C6	0.0443 (8)	0.0584 (11)	0.0391 (8)	−0.0062 (7)	0.0114 (7)	−0.0101 (7)
C7	0.0399 (8)	0.0443 (9)	0.0388 (8)	−0.0035 (6)	0.0136 (6)	−0.0057 (6)
C8	0.0420 (8)	0.0426 (9)	0.0453 (8)	−0.0053 (6)	0.0155 (7)	−0.0073 (7)
C9	0.0401 (8)	0.0377 (8)	0.0450 (8)	0.0004 (6)	0.0096 (7)	−0.0021 (6)
C10	0.0447 (8)	0.0387 (8)	0.0551 (10)	0.0038 (7)	0.0160 (7)	0.0014 (7)
C11	0.0621 (11)	0.0437 (10)	0.0633 (11)	0.0079 (8)	0.0129 (9)	0.0098 (8)
C12	0.0504 (11)	0.0565 (12)	0.0786 (14)	0.0129 (9)	−0.0007 (10)	−0.0018 (10)
C13	0.0374 (9)	0.0718 (13)	0.0878 (15)	−0.0009 (9)	0.0126 (9)	−0.0096 (12)
C14	0.0462 (9)	0.0581 (11)	0.0640 (11)	−0.0077 (8)	0.0191 (8)	−0.0053 (9)

### Geometric parameters (Å, °)

**Table:**

O3—H3A	0.8200	C8—O2	1.211 (2)
C1—O1	1.1967 (19)	C8—N1	1.391 (2)
C1—N1	1.407 (2)	C9—C14	1.376 (2)
C1—C2	1.485 (2)	C9—C10	1.385 (2)
C2—C3	1.370 (2)	C9—N1	1.431 (2)
C2—C7	1.382 (2)	C10—O3	1.351 (2)
C3—C4	1.390 (2)	C10—C11	1.385 (2)
C3—H3	0.9300	C11—C12	1.375 (3)
C4—C5	1.392 (3)	C11—H11	0.9300
C4—Cl1	1.7213 (18)	C12—C13	1.375 (3)
C5—C6	1.381 (3)	C12—H12	0.9300
C5—Cl2	1.7229 (17)	C13—C14	1.377 (3)
C6—C7	1.381 (2)	C13—H13	0.9300
C6—H6	0.9300	C14—H14	0.9300
C7—C8	1.475 (2)		
			
C10—O3—H3A	109.5	C6—C7—C8	130.28 (15)
C8—N1—C1	111.65 (13)	C2—C7—C8	108.41 (14)
C8—N1—C9	123.59 (13)	O2—C8—N1	124.64 (16)
C1—N1—C9	124.56 (13)	O2—C8—C7	129.15 (16)
O1—C1—N1	125.34 (15)	N1—C8—C7	106.20 (13)
O1—C1—C2	129.33 (15)	C14—C9—C10	120.68 (16)
N1—C1—C2	105.33 (13)	C14—C9—N1	119.67 (16)
C3—C2—C7	121.90 (15)	C10—C9—N1	119.64 (14)
C3—C2—C1	129.71 (15)	O3—C10—C11	123.37 (17)
C7—C2—C1	108.32 (14)	O3—C10—C9	117.63 (15)
C2—C3—C4	117.19 (16)	C11—C10—C9	119.00 (16)
C2—C3—H3	121.4	C12—C11—C10	120.09 (19)
C4—C3—H3	121.4	C12—C11—H11	120.0
C3—C4—C5	121.12 (16)	C10—C11—H11	120.0
C3—C4—Cl1	118.46 (14)	C13—C12—C11	120.52 (18)
C5—C4—Cl1	120.42 (13)	C13—C12—H12	119.7
C6—C5—C4	121.06 (15)	C11—C12—H12	119.7
C6—C5—Cl2	118.48 (14)	C12—C13—C14	119.85 (18)
C4—C5—Cl2	120.46 (14)	C12—C13—H13	120.1
C7—C6—C5	117.47 (16)	C14—C13—H13	120.1
C7—C6—H6	121.3	C9—C14—C13	119.84 (19)
C5—C6—H6	121.3	C9—C14—H14	120.1
C6—C7—C2	121.24 (16)	C13—C14—H14	120.1
			
O1—C1—C2—C3	−4.3 (3)	C14—C9—C10—O3	179.76 (17)
N1—C1—C2—C3	175.38 (16)	N1—C9—C10—O3	0.7 (2)
O1—C1—C2—C7	178.66 (16)	C14—C9—C10—C11	−0.2 (3)
N1—C1—C2—C7	−1.70 (17)	N1—C9—C10—C11	−179.21 (16)
C7—C2—C3—C4	1.2 (2)	O3—C10—C11—C12	−178.91 (19)
C1—C2—C3—C4	−175.57 (16)	C9—C10—C11—C12	1.0 (3)
C2—C3—C4—C5	−1.0 (3)	C10—C11—C12—C13	−1.0 (3)
C2—C3—C4—Cl1	178.97 (12)	C11—C12—C13—C14	0.1 (3)
C3—C4—C5—C6	0.0 (3)	C10—C9—C14—C13	−0.7 (3)
Cl1—C4—C5—C6	179.98 (13)	N1—C9—C14—C13	178.34 (17)
C3—C4—C5—Cl2	179.64 (13)	C12—C13—C14—C9	0.7 (3)
Cl1—C4—C5—Cl2	−0.3 (2)	O2—C8—N1—C1	177.64 (16)
C4—C5—C6—C7	0.9 (2)	C7—C8—N1—C1	−3.01 (17)
Cl2—C5—C6—C7	−178.74 (13)	O2—C8—N1—C9	−7.3 (3)
C5—C6—C7—C2	−0.8 (2)	C7—C8—N1—C9	172.01 (14)
C5—C6—C7—C8	175.66 (16)	O1—C1—N1—C8	−177.39 (15)
C3—C2—C7—C6	−0.3 (2)	C2—C1—N1—C8	2.95 (17)
C1—C2—C7—C6	177.09 (15)	O1—C1—N1—C9	7.6 (3)
C3—C2—C7—C8	−177.42 (15)	C2—C1—N1—C9	−172.02 (14)
C1—C2—C7—C8	−0.07 (17)	C14—C9—N1—C8	−102.12 (19)
C6—C7—C8—O2	4.3 (3)	C10—C9—N1—C8	76.9 (2)
C2—C7—C8—O2	−178.86 (17)	C14—C9—N1—C1	72.3 (2)
C6—C7—C8—N1	−174.99 (17)	C10—C9—N1—C1	−108.70 (18)
C2—C7—C8—N1	1.84 (17)		

### Hydrogen-bond geometry (Å, °)

**Table:**

*D*—H···*A*	*D*—H	H···*A*	*D*···*A*	*D*—H···*A*
O3—H3A···O2^i^	0.82	1.90	2.7235 (18)	177
C3—H3···O3^ii^	0.93	2.55	3.397 (2)	152
C13—H13···O3^iii^	0.93	2.59	3.505 (2)	168

Symmetry codes: (i) *x*, −*y*+3/2, *z*−1/2; (ii) −*x*+1, −*y*+1, −*z*+1; (iii) *x*−1, *y*, *z*.
